# Resveratrol sequentially induces replication and oxidative stresses to drive p53-CXCR2 mediated cellular senescence in cancer cells

**DOI:** 10.1038/s41598-017-00315-4

**Published:** 2017-03-16

**Authors:** Boxuan Li, Dong Hou, Haiyang Guo, Haibin Zhou, Shouji Zhang, Xiuhua Xu, Qiao Liu, Xiyu Zhang, Yongxin Zou, Yaoqin Gong, Changshun Shao

**Affiliations:** 1 Key Laboratory of Experimental Teratology, Ministry of Education/Department of Molecular Medicine and Genetics, Shandong University School of Medicine, Jinan, Shandong 250012 China; 2 Department of Genetics/Human Genetics Institute of New Jersey, Rutgers University, Piscataway, NJ 08854 USA

## Abstract

Resveratrol (RSV) acts either as an antioxidant or a pro-oxidant depending on contexts. RSV-treated cancer cells may experience replication stress that can lead to cellular senescence or apoptosis. While both oxidative and replication stresses may mediate the anti-proliferation effect of RSV, to what extent each contributes to the impaired proliferation in response to RSV remains uncharacterized. We here report the study of the roles of replication and oxidative stresses in mediating cellular senescence in cancer cells treated with RSV. RSV induced S-phase arrest and cellular senescence in a dose-dependent manner in U2OS and A549 cancer cells as well as in normal human fibroblasts. We observed that nucleosides significantly alleviated RSV-induced replication stress and DNA damage response, and consequently attenuating cellular senescence. While the elevation of reactive oxygen species (ROS) also mediated the pro-senescent effect of RSV, it occurred after S-phase arrest. However, the induction of ROS by RSV was independent of S-phase arrest and actually reinforced the latter. We also demonstrated a critical role of the p53-CXCR2 axis in mediating RSV-induced senescence. Interestingly, CXCR2 also functioned as a barrier to apoptosis. Together, our results provided more insights into the biology of RSV-induced stress and its cellular consequences.

## Introduction

Resveratrol (RSV), a natural phytoalexin found in red wine, peanuts, berries and many other botanicals, has been shown to have many health benefits including cancer chemoprevention and protection against metabolic diseases caused by high-calorie diet^1–3^. Cells exposed to RSV may experience replication stress, as reflected by S-phase arrest, and eventually undergo apoptosis or senescence^4–13^. However, RSV can also suppress senescence in certain contexts^14, 15^. It is of particular note that RSV possesses both antioxidant and pro-oxidant properties depending on the cell types it is applied to, dose applied and other experimental conditions^12, 16–22^. Its function as an antioxidant is attributed to its abilities to directly scavenge the oxygen radicals and to downregulate NADPH oxidases^16–18, 21, 22^. However, RSV also increases oxidative stress by upregulating NADPH oxidases and consequently causes cellular senescence^12, 20^.

S-phase arrest, which is commonly observed in cells treated with RSV^4–11, 23^, reflects replication stress. Because replication forks can be stalled at oxidized nucleotides^24, 25^, the S-phase arrest caused by RSV could be attributed to increased oxidative stress. However, RSV can also inhibit ribonucleotide reductase^26^ and DNA polymerases^27^, which are critical for the progression of DNA synthesis. Whether RSV-induced replication stress is also mediated by the inhibition of ribonucleotide reductase has not been tested. Persistence of replication stress, as caused by inappropriate activation of oncogenes, is a cause of cellular senescence in normal cells^28, 29^. Therefore, while it is known that RSV can cause senescence, the roles of oxidative and replication stresses in mediating such effect remain to be clarified. Moreover, the factors that determine the cellular fate of apoptosis or senescence under RSV treatment have not been characterized.

In this report, we address the role of replication and oxidative stresses in mediating cellular senescence of cancer cells treated with RSV. We find that RSV sequentially induces replication and oxidative stresses. While replication stress occurs prior to the onset of oxidative stress, it is reinforced by the latter. We show that p53-regulated CXCR2 plays a critical role in mediating RSV-induced senescence and functions as a barrier to apoptosis.

## Results

### RSV induces S phase arrest, DNA damage response and cellular senescence

**Figure 1:**
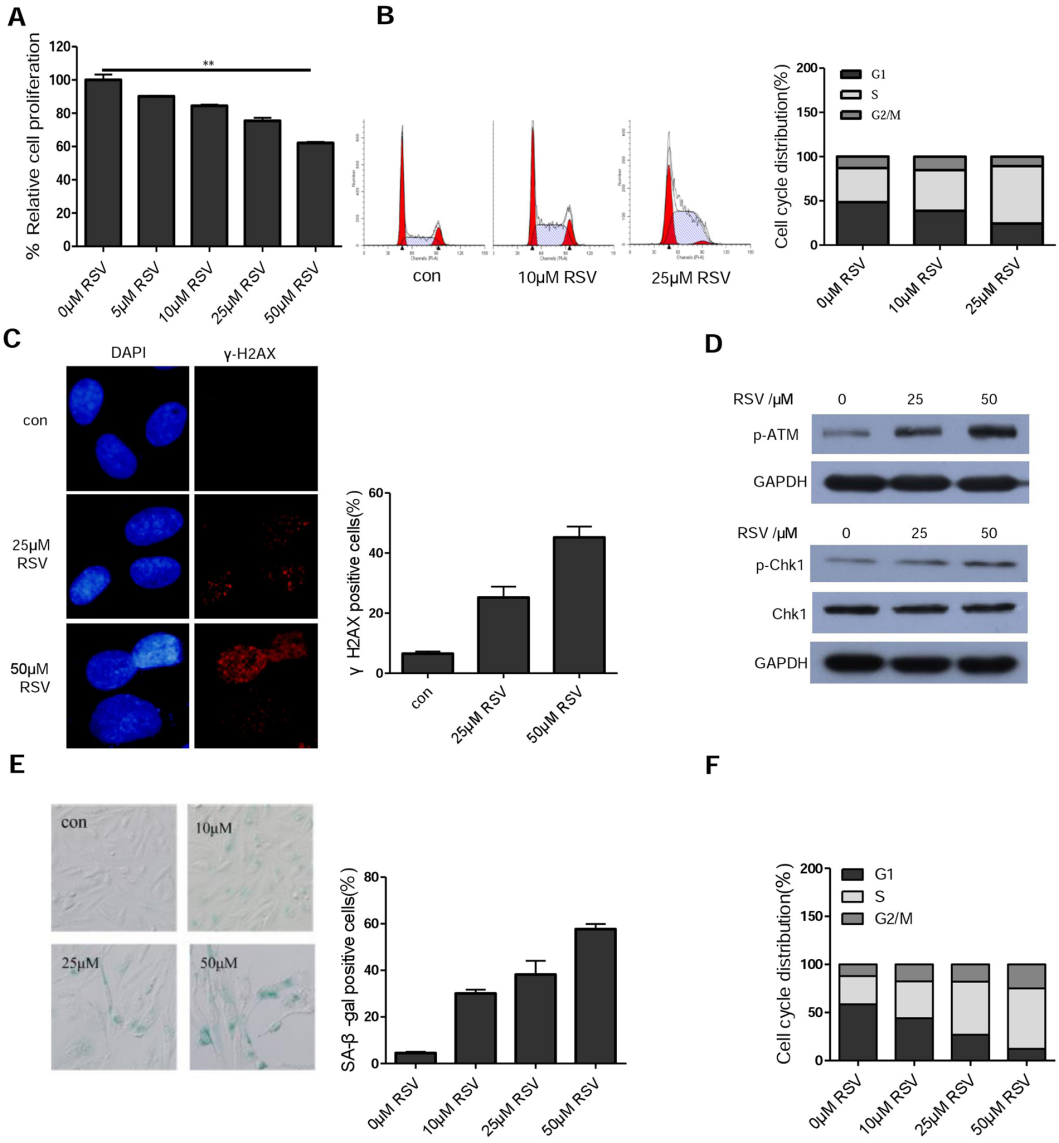
Resveratrol induces S phase arrest, DDR and cellular senescence. (**A**) Cell viability determined by MTT assay. U2OS cells were treated with the indicated concentrations of resveratrol (RSV) for 48 h, and cell viability was determined by MTT. **P < 0.01, when compared with control group. (**B**) RSV induces S-phase arrest of U2OS cells. Cells were treated with different concentration of RSV for 24 h and then cell cycle distribution was measured by FACS analysis. Data are presented as the mean ± SD of values from triplicate experiments. (**C**) Detection of γ-H2AX by immunofluorescence. Cells were treated with the indicated concentrations of RSV for 24 h, stained with mouse anti-γ-H2AX(Ser139) antibody and goat anti-mouse TRITC-conjugated secondary antibody, then counterstained with DAPI (blue). (**D**) Western blotting analysis of p-ATM, p-Chk1 protein levels in U2OS cells treated with different concentration of resveratrol for 24 h. (**E**) Induction of cellular senescence by RSV. U2OS cells were treated without or with different concentration of RSV for 7 days. Cellular senescence was examined by SA-β-gal staining. (**F**) Cell cycle distribution of U2OS cells after being treated with different concentration of RSV for 5 days.

We first subjected human osteosarcoma cells U2OS to various concentrations of RSV for 48 h and determined their effect on cell viability. As shown in Fig. 1A, RSV inhibited the proliferation of U2OS cells in a dose-dependent manner. Analysis of cell cycle distribution by flow cytometry indicated that RSV dose-dependently induced S-phase arrest (Fig. 1B). There was a remarkable increase in the amount of γ-H2AX, a marker for DNA double-strand breaks, in RSV-treated cells, as revealed by immunofluorescence (Fig. 1C). Meanwhile, ATM, a master regulator of DNA damage response, was activated by RSV (Fig. 1D). In addition, typically of cells experiencing replication stress, the level of phosphorylated CHK1 level was increased (Fig. 1D). Consistent with previous report in HCT116 colon cancer cells^12^, there was a significant induction of cellular senescence (Fig. 1E). It appeared that the cells arrested at S-phase could progress to senescence, as the majority of cells remained in S-phase when they became senescent (Fig. 1F).

It should be noted that RSV induced S-phase arrest and senescence in U2OS only at relatively high concentrations (≥10 µM) (Fig. 1B,E). Similarly, while RSV at 25 and 50 µM induced S-phase arrest and senescence in normal human fibroblasts (NHFs) and A549 cells, it had not such effect at 5 µM (Fig. S1A,B). Because our findings were seemingly contradictory to previous reports showing that RSV could inhibit senescence caused by other stressors^14, 15, 30, 31^, we also tested the effect of RSV on cellular senescence caused by H_2_O_2_ in U2OS and NHF cells. As shown in Fig. S1C, RSV could indeed attenuate H_2_O_2_-induced senescence, though the dose required for such an effect differed in the two types of cells. These results suggest that whether RSV acts as an inducer or inhibitor of cellular senescence is context-dependent.

### RSV induced cellular senescence is attenuated by nucleosides

**Figure 2:**
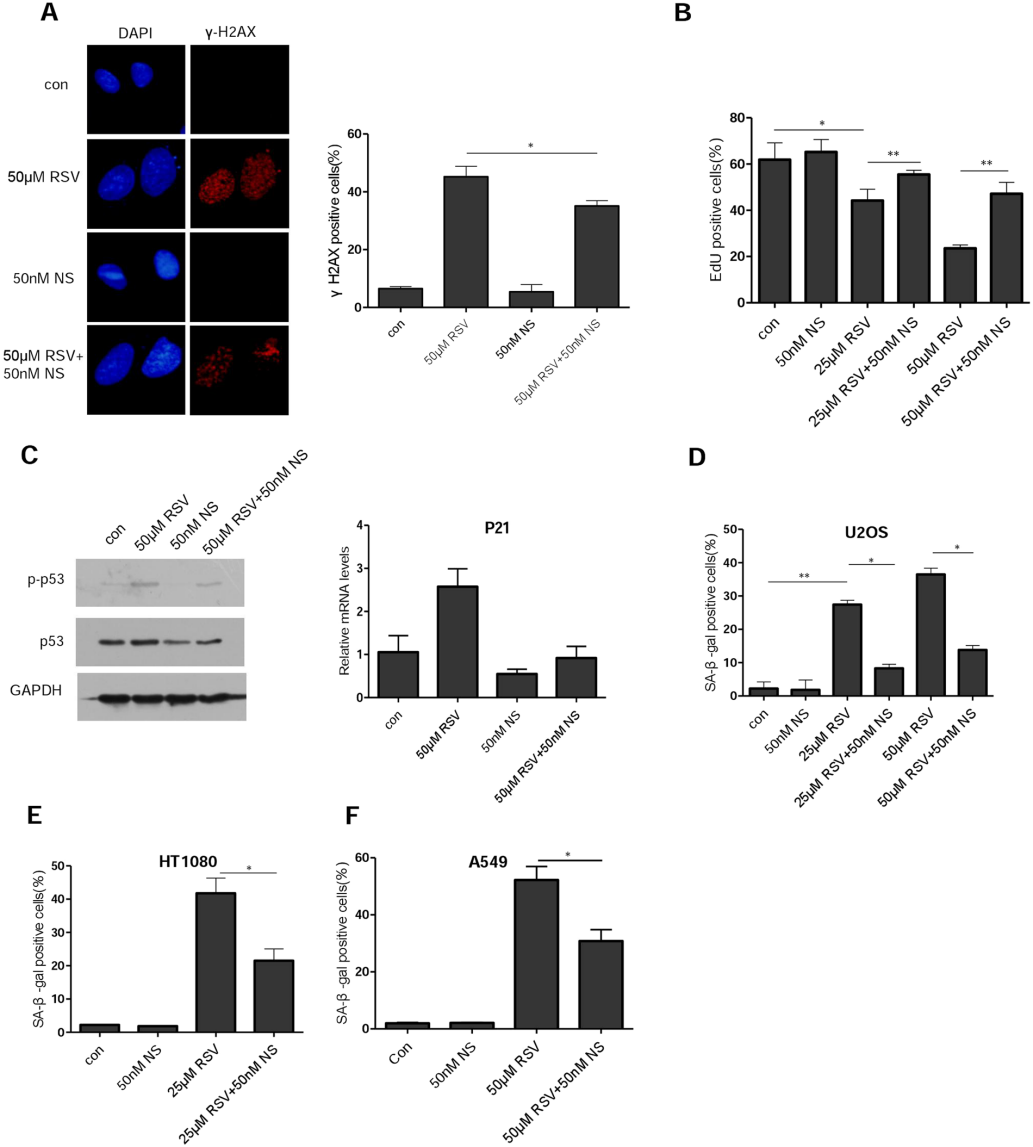
Resveratrol-induced cellular senescence can be attenuated by nucleosides. (**A**) Immunofluorescence staining of γ-H2AX in U2OS cells. Cells growing on coverslips in 6-well plates were exposed to RSV (50 μM) alone or in combination with nucleosides (50 nM) for 24 h, and fixed for examination by immunofluorescence. (**B**) EdU proliferation assay was performed in U2OS cells 48 h after treatment with RSV alone or in combination with nucleosides, the percentages of EdU positive cells are shown as the mean ± S.D. from three independent experiments. (**C**) Left, U2OS cells were exposed to RSV with or without nucleosides for 48 h. Whole cell lysates were analyzed by immunoblotting with antibodies specific for p-p53 and p53, respectively. GAPDH was used as a loading control. Right, with the same treatment as in the left, but examined for expression of p21 mRNA levels by RT-PCR. (**D**) U2OS cells were incubated with RSV with or without addition of the indicated concentration of nucleosides (NS). On day 7, cells were examined for SA-β-gal activity. Mean of three independent experiments with SEM is shown. (**E**,**F**) The same as in (**D**) but in HT1080 and A549 cells, respectively. *P < 0.05, **P < 0.01.

RSV was reported to function as an inhibitor of ribonucleotide reductase and to be more potent than hydroxyurea, a commonly used inducer of replication stress, in inhibiting the activity of mouse ribonucleotide reductase^26^. Interestingly, suppression of nucleotide reductase also underlies the establishment and maintenance of oncogene-induced senescence, which can be rescued by exogenous nucleosides^32^. We speculated that exogenous nucleosides may similarly rescue RSV-induced replication stress and senescence if RSV operates by inhibiting ribonucleotide reductase. We tested this and indeed found that induction of DNA damage by RSV was significantly attenuated by nucleosides (Fig. 2A). Furthermore, nucleosides greatly rescued the stalled replication, as shown by the increased EdU incorporation (Fig. 2B). In keeping with the reduction in DNA damage, p53 activation was attenuated by nucleosides (Fig. 2C). Importantly, RSV-induced senescence was greatly reduced by nucleosides (Fig. 2D). We tested the inhibitory effect of nucleosides on RSV-induced senescence in two additional cell lines HT1080 (fibrosarcoma) and A549 (lung cancer) and obtained similar results (Fig. 2E,F). These results suggest that replication stress mediates RSV-induced senescence and the senescence can be effectively rescued by exogenous nucleosides.

### RSV sequentially induces replication and oxidative stresses

**Figure 3:**
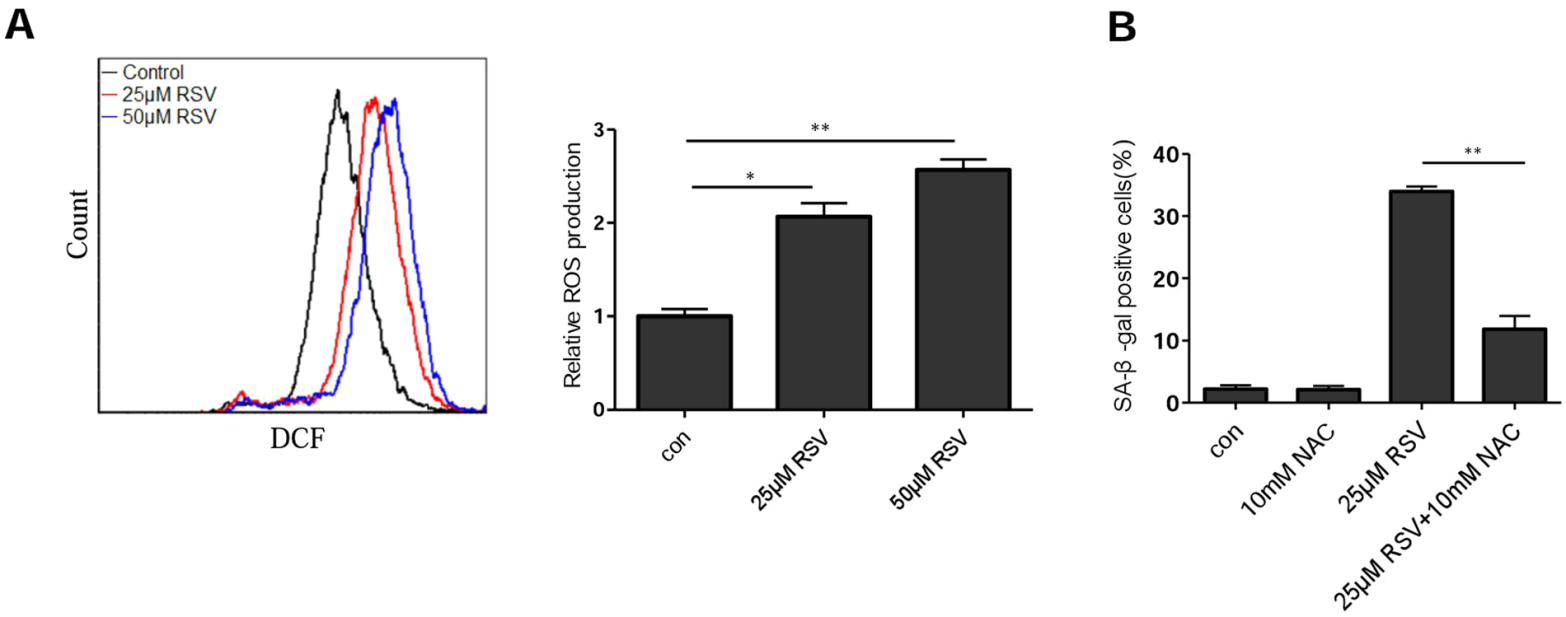
Resveratrol-induced cellular senescence can be attenuated by NAC. (**A**) U2OS cells were treated with different concentration of RSV for 48 h. ROS was measured by flow cytometry. Data summary was shown on the right, Mean of three independent experiments with SEM is shown. *P < 0.05, **P < 0.01. (**B**) NAC attenuates RSV induced senescence. U2OS cells were incubated with RSV with or without addition of the NAC, on day 7, cells were examined for SA-β-gal activity.

**Figure 4:**
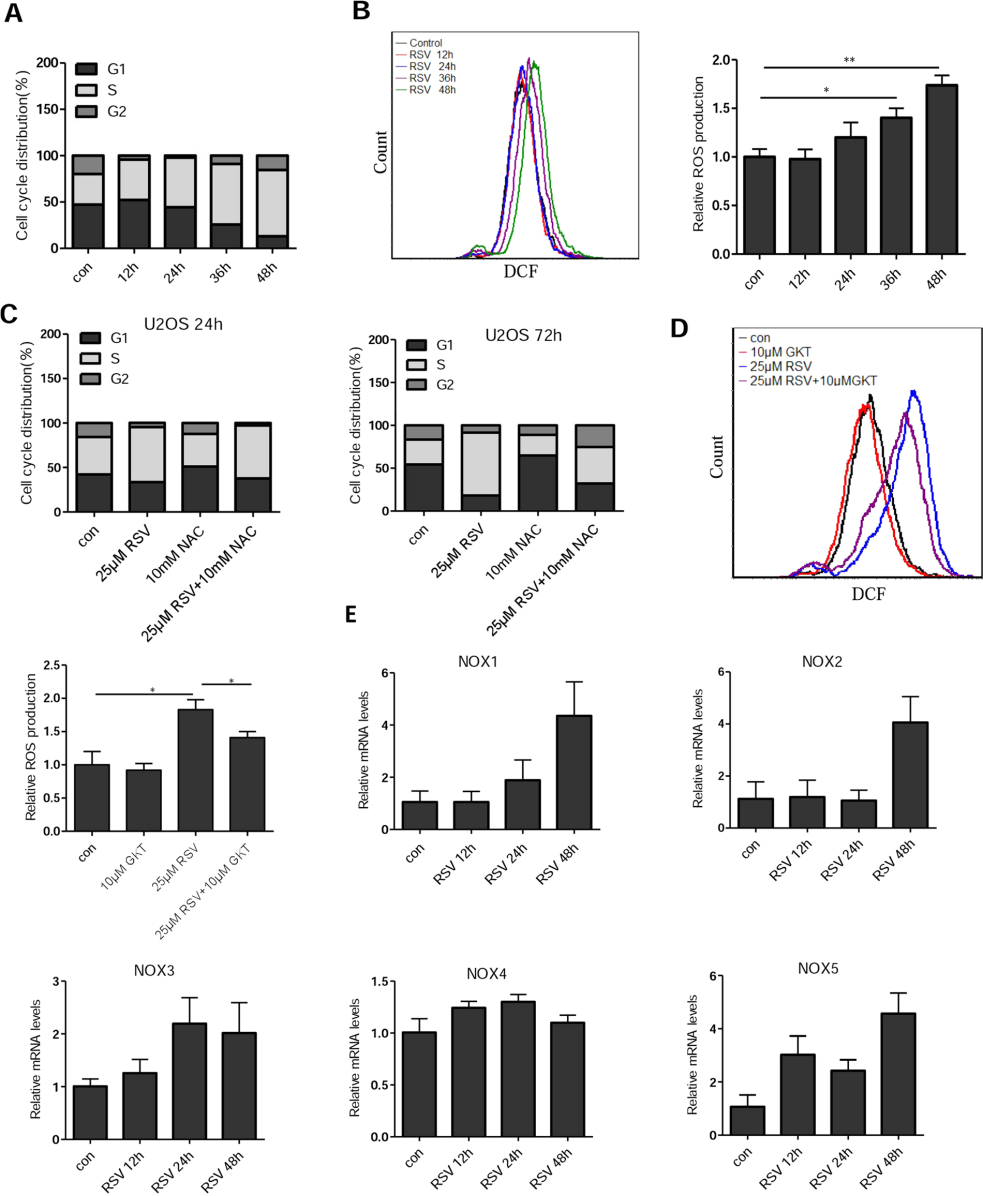
S-phase arrest precedes onset of oxidative stress. Cell cycle distribution (**A**) and ROS levels (**B**) of U2OS cells were measured at different time points after RSV treatment. Cells were treated with 25 μM RSV for 0, 12, 24, 36, and 48 h, then the cell cycle distribution and ROS levels were measured by FACS analysis. (**C**) U2OS cells were pretreated with or without 10 mM NAC for 1 h, and then with or without 25 μM RSV for 24 h or 72 h. (**D**) Effect of GKT137831(GKT), a NADPH oxidase NOX1/NOX4 inhibitor, on ROS production. U2OS cells were incubated with RSV with or without GKT for 48 h and then ROS was measured by FACS analysis. *P < 0.05. (**E**) qRT-PCR analysis of various human NOX genes. U2OS cells were treated with RSV (50 μM) for different durations, the expression levels of NOX mRNAs were determined using RT-PCR.

Oxidative stress was also shown to be a key mediator of RSV-induced senescence in HCT116 colon cancer cells and human endothelial cells^12, 20^. We next measured the level of ROS in U2OS cells after they were treated with RSV. Indeed, the ROS levels were significantly elevated by RSV (Fig. 3A). Importantly, RSV-induced senescence was significantly attenuated by NAC (Fig. 3B). Thus, as in other types of human cells, oxidative stress also mediates RSV-induced senescence. Since both replication and oxidative stresses mediate RSV-induced senescence, we next examined the kinetics of two types of stress following RSV treatment. While S-phase arrest was noticeable at 12 h (Fig. 4A), elevation of ROS was detected only at 36 h after RSV treatment (Fig. 4B). Furthermore, co-treatment with NAC for 24 h was unable to rescue the S-phase arrest, though a noticeable rescuing effect was detected at 72 h (Fig. 4C). Similarly, S-phase arrest was evident at 24 h after HT1080 cells were treated with RSV (Fig. S2A), but an elevation in ROS level was detected only at 36 h (Fig. S2B). As in U2OS cells, NAC was unable to rescue S-phase arrest caused by RSV only at 24 h (Fig. S2C). These results indicate that S-phase arrest occurs before the increase in ROS.

Upregulation of NADPH oxidases 1 and 4 by RSV was reported to be responsible for senescence in human endothelial cells^20^. To test whether NOX1/4 is also responsible for the increased oxidative stress in RSV-treated U2OS cells, we treated the cells with NOX1/4 inhibitor GKT137831(GKT). As shown in Fig. 4D, GKT significantly attenuated the induction of ROS by RSV, but did not completely abolish the pro-oxidant effect of RSV. We next determined the expression of genes encoding various NADPH oxidases by quantitative PCR and observed a significant increase in NOX1 expression at 48 h, but not at 24 h (Fig. 4E). In addition, NOX2, NOX3 and NOX5 appeared to be upregulated by RSV, especially at 48 h. NOX4, however, remained steady during the duration of RSV treatment. Together, these results suggest that oxidative stress as a mediator of cellular senescence occurs after cells already experience replication stress.

We then tested whether RSV-induced oxidative stress is dependent on S-phase arrest. To this end, we arrested NHFs at G0/G1 phase by serum starvation for 48 h and then applied RSV (Fig. S3A). As shown in Fig. S3B, the ROS levels were elevated by RSV in serum-starved cells, indicating that the ROS increase by RSV is not specific to S-phase arrested cells. There was also a significant increase in DNA damage, as reflected the increased percentage of γ-H2AX positive cells (Fig. S3C). The partial rescue of S-phase arrest by prolonged treatment of NAC (Fig. 4C) also suggests that while the ROS increase is a relatively delayed response when compared to S-phase arrest, it may still function to reinforce or maintain replication stress in RSV-treated cells.

However, the pro-oxidant effect of RSV was dependent on its concentration in U2OS as well as in NHFs and A549 cells (Fig. S4A–C). Consistent with previous reports of RSV acting as an antioxidant^33, 34^, when NHF and U2OS cells were stressed by H_2_O_2_, RSV could actually reduce the level of ROS, dose-dependently in NHF, but with oscillations in U2OS cells (Fig. S4D,E). These results suggest that RSV may either act as an antioxidant or pro-oxidant depending on contexts.

### p53-mediated CXCR2 upregulation contributes to cellular senescence

**Figure 5:**
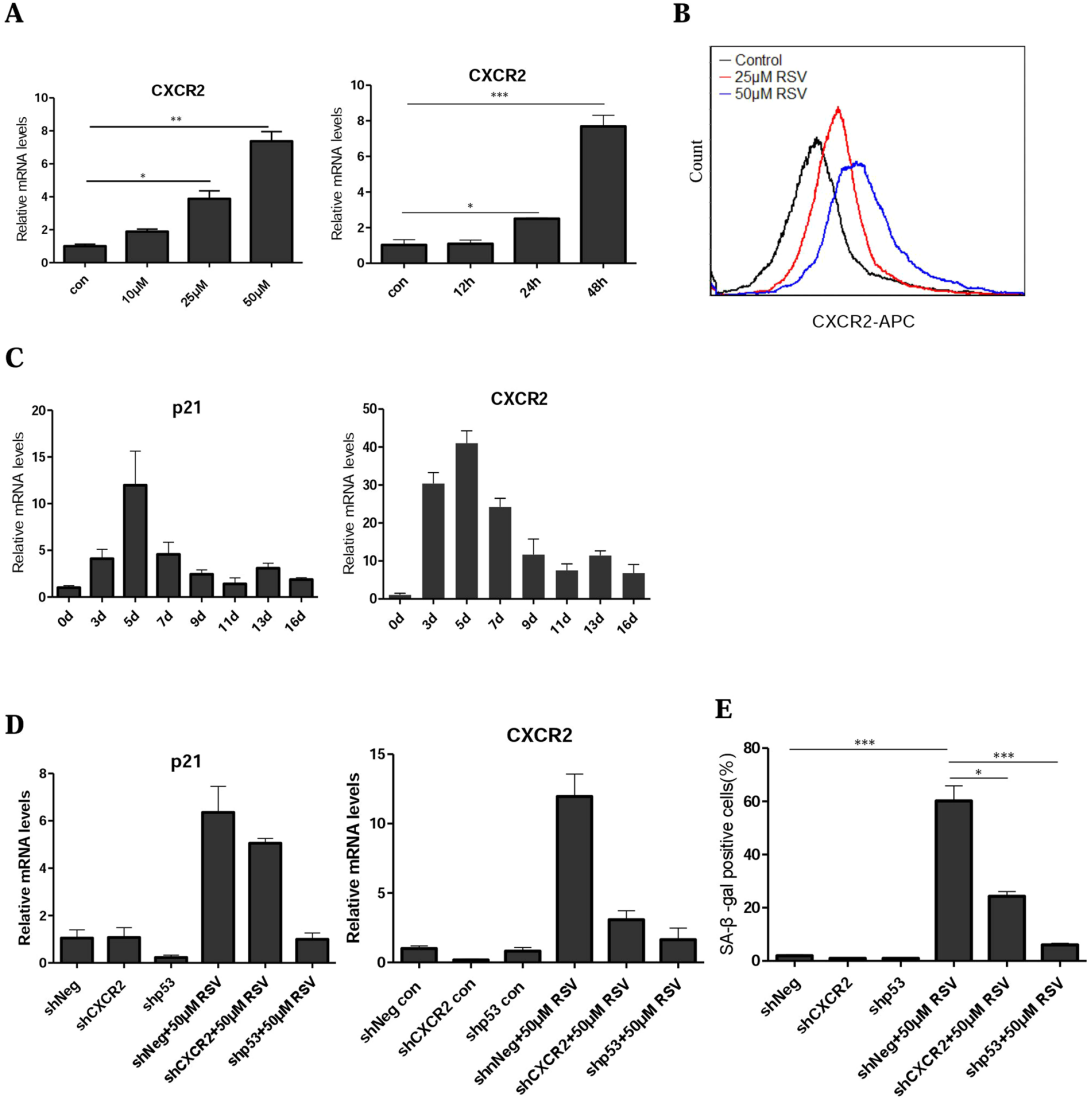
Upregulation of p53-CXCR2 axis mediates cellular senescence. (**A**) CXCR2 mRNA was determined using RT-PCR. U2OS cells were treated with different concentration of RSV for 48 h (left) and 25 μM RSV for 12, 24 and 48 h (right). (**B**) Detection of CXCR2 by flow cytometry using mouse anti-human CD182 antibody. Cells were treated with RSV (25 and 50 μM) for 48 h. (**C**) p21 and CXCR2 mRNAs were determined using RT-PCR. U2OS cells were treated with 25 μM RSV for the indicated days. (**D**) The mRNA levels of CXCR2, p21 in shNeg, shp53 and shCXCR2 U2OS cells were measured 48 h after treatment with RSV (50 μM). (**E**) The cells were treated the same as (**D**), on day 7, cellular senescence was examined by SA-β-gal staining.

RSV-induced senescence requires the activation of p53^12^. We previously showed that the upregulation of p53-CXCR2 axis plays a key role in stress-induced senescence in normal human fibroblasts and U2OS cells^35^. Indeed, CXCR2 transcription was significantly increased by RSV in a dose- and time-dependent manner (Fig. 5A). The increased expression of CXCR2 was confirmed by flow cytometry analysis of CXCR2 (CD182) (Fig. 5B). However, after CXCR2 expression reached its peak level at day 5, it began to subside in the following days (Fig. 5C). p21 followed a similar pattern. Thus, it appeared that once senescence is established, only modest levels of the effector proteins are maintained.

Consistent with the results that RSV can suppress senescence and oxidative stress in cells treated by H_2_O_2_ (Figs S1C and S4D,E), H_2_O_2_-induced CXCR2 expression can be attenuated by RSV (Fig. S5), indicating that while CXCR2 mediates RSV-induced cellular senescence at higher doses, its expression was attenuated by RSV under a different condition.

We confirmed that the upregulation of CXCR2 following RSV treatment was dependent on p53, since the expression of CXCR2, like that of p21, in response to RSV was greatly reduced when p53 was depleted (Fig. 5D). Importantly, depletion of CXCR2 greatly attenuated RSV-induced senescence (Fig. 5E). Together, these results indicate that the p53-CXCR2 axis also functions to drive RSV-induced senescence.

### CXCR2 protects cells from stress-induced apoptosis

**Figure 6:**
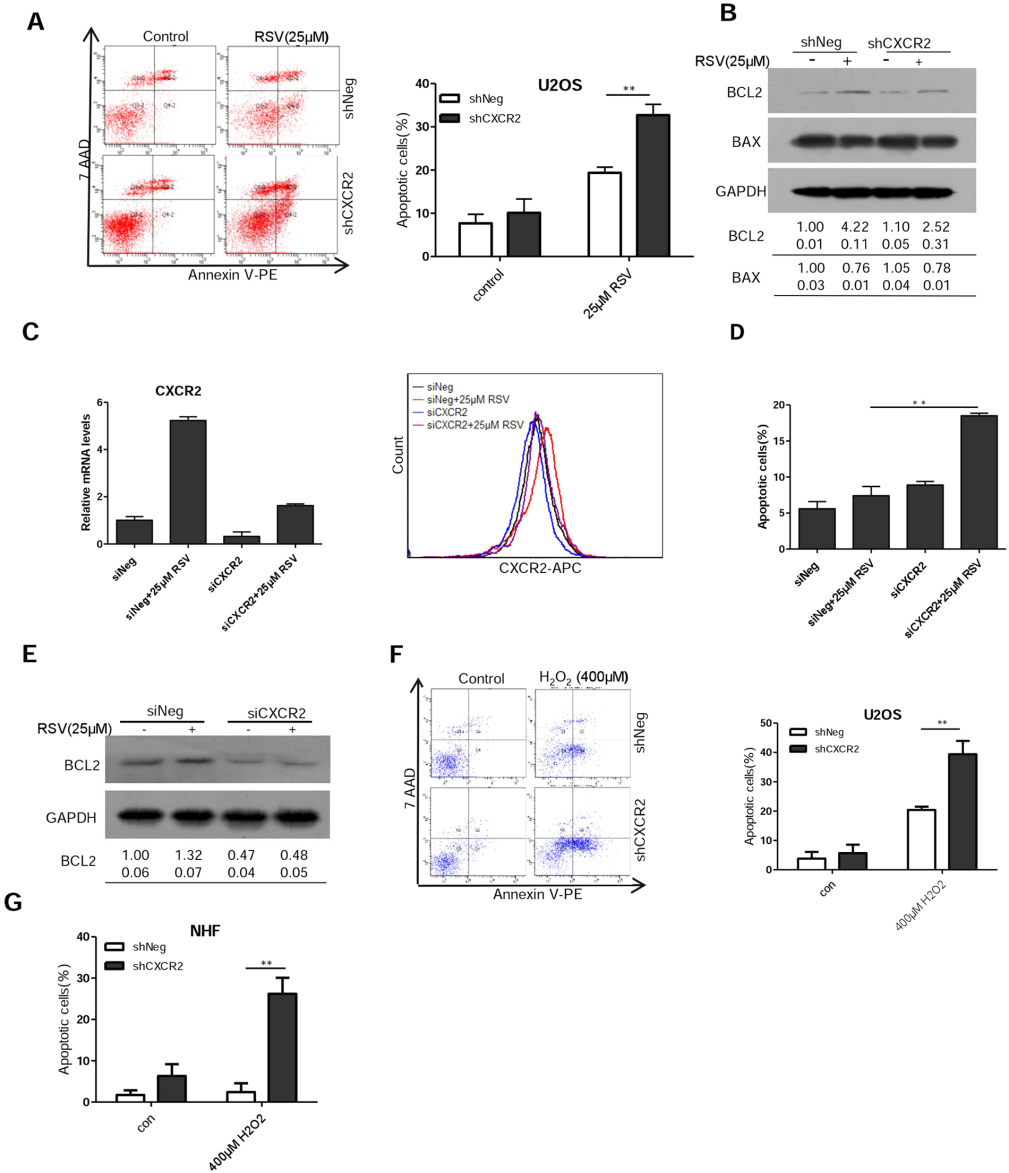
CXCR2 protects cells from undergoing stress-induced apoptosis. (**A**) Apoptosis in shNeg and shCXCR2 U2OS cells was measured 3 days after treatment with RSV (25 μM) by flow cytometry. (**B**) Cells were treated as in (**A**) and whole-cell extracts were collected for Western blot analysis using BCL2 and BAX antibodies. (**C**) Downregulation of CXCR2 by siRNA as measured by RT-PCR and flow cytometry. U2OS cells were transfected with siRNA duplexes (200 nmol/L) specific to CXCR2 or scrabbled oligo in serum-free medium for 6 hours, then were incubated with complete medium for 24 h and then incubated with RSV for 3 days. (**D**) U2OS cells were treated the same as in (**C**) and apoptosis was measured by flow cytometry. (**E**) The U2OS cells were treated the same as in (**C**) and whole-cell extracts were collected for Western blot analysis using BCL2 antibodies. (**F**) Apoptosis in shNeg and shCXCR2 U2OS cells was measured by flow cytometry 2 days after treatment with H_2_O_2_ (400 μM). Results shown are representative of three independent experiments. (**G**) The shNeg and shCXCR2 NHF cells were treated the same as in (**F**) and apoptosis was measured by flow cytometry. The numbers shown below Western blot images are means (first row) and SE (second row) of band intensities relative to control. Signals on the immunoblots were analyzed by ImageJ, normalized with that of GAPDH.

While the cancer cells we studied here primarily undergo senescence when treated with RSV, apoptosis was the main fate for MCF-7 breast cancer cells and LNCaP prostate cancer cells^10, 11^. Because senescent cells are intrinsically resistant to apoptosis^36–38^. We hypothesized that by driving cellular senescence CXCR2 might function as a barrier to apoptosis. We therefore measured the rate of apoptosis in shCXCR2 and control cells (shNeg) in response to RSV. Indeed, the percentage of apoptotic cells was significantly higher in shCXCR2 cells than that in shNeg cells when treated with RSV (Fig. 6A). Consistently, the induction of anti-apoptotic protein BCL2 by RSV was attenuated with the depletion of CXCR2 (Fig. 6B). Depletion of CXCR2 by siRNA similarly caused more U2OS cells to undergo apoptosis when compared to control (Fig. 6C,D). As in shCXCR2 cells, BCL2 expression was also decreased in U2OS cells treated with siRNA against CXCR2 (Fig. 6E). CXCR2 knockdown also rendered U2OS cells and normal human fibroblasts more prone to apoptosis induced by hydrogen peroxide (Fig. 6F,G). Together, these results indicate that by driving cellular senescence, CXCR2 effectively protects cells from apoptosis.

## Discussion

RSV has been reported to modulate many biological processes and to change a large variety of phenotypes at cellular and organismal levels. It has been reported to act either as an antioxidant or pro-oxidant and consequently having either protective or deleterious effect on its target cells. We here showed that RSV can induce cellular senescence of cancer cells by increasing both replication and oxidative stresses. Nucleosides and antioxidant can each attenuate RSV-induced cellular senescence, which was accompanied by decrease of DNA damage and reduction of p53 activation. Whereas both replication and oxidative stresses mediate RSV-induced senescence, the two acted sequentially and oxidative stress appeared to occur at a later stage than replication stress. However, oxidative stress also functioned to reinforce S-phase arrest in RSV-treated cells since application of antioxidant NAC can effectively rescue the arrest. We previously reported a critical role of p53-CXCR2 axis in stress-induced cellular senescence^35^. Similarly, the upregulation of p53-CXCR2 axis is largely responsible for RSV-induced senescence. Importantly, by driving cellular senescence, CXCR2 appeared to function as a barrier to apoptosis.

The prosenescent effect of RSV on osteosarcoma cells we described here is consistent with previous reports of RSV as an inducer of senescence of p53-positive cancer cells and endothelial cells^12, 13, 20, 39^. RSV is known to improve cellular function as an activator of SIRT1 and PGC-1α^3, 40^. It appears that whether RSV exerts pro-senescent or anti-senescent effect is also related to its function as pro-oxidant or antioxidant. While its pro-senescent effect is usually mediated by its induction of oxidative stress, it tends to function as an antioxidant where it protects cells from undergoing senescence. However, RSV conferring a protective effect by functioning as an antioxidant was usually observed in cells stressed by other insults^18, 21, 22, 31^. Therefore, the pro- or anti-oxidant function of RSV is context-dependent.

Our results showed that replication stress could contribute to RSV-induced senescence independent of oxidative stress. It occurs earlier than the elevation of ROS and the RSV-induced replication arrest and senescence can be rescued by nucleosides. However, in human endothelial cells treated with RSV, the S-phase arrest appeared to be solely mediated by oxidative stress^20^. Nevertheless, oxidative stress indeed played an important role in sustaining S-phase arrest in RSV-treated cancer cells because prolonged treatment with antioxidant NAC was eventually able to alleviate S-phase arrest. It should also be noted that the NADPH oxidases that mediate oxidative stress vary between cell types. While NOX1 and NOX4 were shown to be responsible for oxidative stress in human endothelial cells^20^, NOX5 appeared to drive senescence in RSV-treated lung cancer cells^13^.

Our study also revealed an important role of CXCR2 in driving RSV-induced cellular senescence. Apoptosis and cellular senescence are mutually exclusive cell fates and the BCL2 family of anti-apoptotic factors are required for the survival of senescent cells^36–38^. We showed that while anti-apoptotic factor BCL2 could be induced by RSV, its expression was reduced when CXCR2 was depleted, suggesting that CXCR2 may promote cell survival and decrease apoptosis via BCL2. Interestingly, increased expression of CXCR2 in ovarian cancer cells was associated with diminished apoptosis^41^. RSV has been documented to induce either apoptosis or senescence in different cancer cell lines. It is possible that the basal and induced levels of CXCR2 and/or BCL2 may determine the choice of those distinct fates.

## Methods

All experiments were carried out in accordance with relevant guidelines and regulations.

### Cell culture and treatment

U2OS, HT1080 and A549 cell lines were obtained from the Cell Bank of Chinese Academy of Sciences (Shanghai). The authenticity of cell lines was characterized at the Cell Bank using DNA markers DXS52, Apo-B, MD17S5 and D2S44. All experiments were performed using cells within 10 passages after receipt. U2OS and NHF cells with stable knockdown of p53 or CXCR2 were as previously described^35^. Resveratrol (RSV) (Sigma, USA) and GKT137831(GKT) (Selleckchem, USA) were dissolved in dimethyl sulfoxide (DMSO). The final concentration of DMSO in the culture medium was less than 0.05% (v/v). Control cultures received the same amount of DMSO. Nucleosides were purchased from Sigma-Aldrich and dissolved in DMSO to make stock solutions (50 mM). N-acetylcysteine (NAC) was purchased from Beyotime Institute of Biotechnology (China). Hydrogen peroxide (H_2_O_2_) was from Sangon Biotech (Shanghai, China). Dulbecco’s Minimum Essential Medium (DMEM), fetal bovine serum, were purchased from Gibco (Carlsbad, CA, USA). The cells were maintained in MEM, or DMEM supplemented with 10% FBS, 100 U/mL penicillin, and 100 mg/mL streptomycin in a humidified 5% CO2/95% air atmosphere at 37 °C.

### Cell viability assay

Cells were plated in 96-well plate the day before RSV treatment. The medium was removed and replaced with fresh medium with or without RSV. Cell density was measured using the MTT following the manufacturer’s instructions. The absorbance of converted dye was measured at the wavelength of 490 nm and the absorbance is directly proportional to cell viability. All experiments were repeated at least three times.

### SA-β-gal staining

Cells were treated with RSV as the indicated, 5 days later, SA-β-gal activity was evaluated using the SA-β -gal staining kit (Beyotime) following the manufacturer’s instructions. At least 500 cells were counted for each sample.

### Cell cycle analysis

Control and treated cells were harvested using 0.25% Trypsin–EDTA, centrifuged (400xg) for 5 min, and washed once with cold PBS. The cells were fixed in 5 ml of cold 70% ethanol at −20 °C overnight. The fixed cells were washed with PBS once and then incubated with 0.5 ml of PBS containing 100 μg/ml RNase (Invitrogen) and 5 μg/ml propidium iodide (Sigma-Aldrich) at room temperature for 30 min. Cell cycle distribution was analyzed by measuring DNA content using a BD Biosciences FACSCan II Analyzer. At least 10,000 cells were collected. For these studies, all experiments were repeated three or more times.

### EdU incorporation

Cells plated on coverslips in 24-well plate were treated as indicated for 48 h, then 5-ethyny-2’-deoxyuridine (EdU) (Cell-Light EdU Cell Proliferation Detection kit, Guangzhou RiboBio) was added at 50 μmol/L and the cells were cultured for an additional 30 min. After the removal of EdU containing medium, the cells were fixed with 4% paraformaldehyde at room temperature for 30 min, washed with glycine (2 mg/mL) for 5 min in a shaker, treated with 0.2% Trion X-100 for 10 mins, washed with PBS twice. Click reaction buffer was then added. After 10 to 30 min, the cells were washed with 0.5% Triton X-100 for three times, stained with DAPI for 10 min at room temperature, washed with 0.5% Triton X-100 for three times, immersed in 150 μL of PBS and examined under a fluorescence microscope.

### Immunofluorescence staining of γ-H2AX

Cells were grown on cover slips and treated with RSV or DMSO (0.01%). After treatment, cells were washed in PBS twice and were fixed in 4% paraformaldehyde for 20 min at room temperature, followed by washing in PBS for three times and treatment with 0.2% Triton X-100 in PBS for 15 min. Cells were then further washed in PBS twice and then blocked with 10% normal goat serum in PBS for 60 min, following which mouse anti-γ-H2AX antibody (Millipore, Billerica, MA) was added at a dilution of 1:200 in 5% normal goat serum in PBS and incubated overnight at 4 °C. Next day, cover slips containing cells were then washed and incubated for one hour at 37 °C in the dark with the Rhodamine-labeled secondary antibody at a dilution of 1:200 in 5% normal goat serum in PBS for 60 min. The secondary antibody solution was then aspirated and the cells were washed four times in PBS. Cells were then incubated in the dark with 4-6-diamidino-2-phenylindole (DAPI) for 5 min and coverslips were mounted with an antifade solution (Molecular Probes, Eugene, OR). Slides were then examined under a fluorescent microscope. For each treatment condition, γ-H2AX foci were counted in at least 100 randomly captured cells.

### Flow cytometry analysis of apoptosis

Apoptotic cells were identified using Annexin V/Dead Cell Apoptosis Kit (Invitrogen). Briefly, cells were treated with RSV or with H_2_O_2_ for 48 h. Thereafter, both adherent and floating cells were harvested, washed twice with ice-cold PBS. Then cells were resuspended in 100 μL of 1 X annexin-binding buffer and incubated at room temperature for 15 mins in the dark with annexin-V- fluorescein isothiocyanate (FITC) and propidium iodide. Cell fluorescence was assessed in a FACScan flow cytometer (Becton Dickinson, San Jose, CA, USA).

### Western blot analysis

Equal amounts (30–50 μg) of protein were separated by 10% SDS-PAGE, transferred to PVDF membrane (Millipore), and blocked with 5% nonfat dry milk in TBS-Tween 20 (0.1%, v/v) for at least 1 h at room temperature. The membrane was incubated with specific primary antibodies at 4 °C for overnight. After washing, the membrane was incubated with the appropriate horseradish peroxidase secondary antibody (diluted 1:5,000; Amersham Pharmacia Biotech) for 1 h. Following three times washes, the blots were developed by ECL kit (Thermo). The antibodies used for Western blot were anti-p-p53 (No. 9284s, Cell Signaling; 1:1000), anti-p53 (sc-126, Santa Cruz; 1:1000), anti-p-CHK1 (ab58567, abcam; 1:1000), anti-CHK1 (No.2360, Cell Signaling Technology; 1:1000),anti-p-ATM (No. 5883s, Cell Signaling Technology; 1:1000), anti-BCL2 (60178-1-Ig, proteintech; 1:1000), anti-BAX (60267-1-lg,proteintech; 1:1000), anti-GAPDH (Chemicon; 1:10000). The protein levels were normalized by GAPDH.

### Quantitative RT-PCR

Quantitative RT-PCR (qRT-PCR) analyses were performed using the Light Cycler 480 sequence Detection System (Roche Applied Science) with SYBR-Green (Invitrogen). The relative levels of gene expression were determined using the cycle threshold method of relative quantification. The following primers were used: NOX1 sense 5′-GCACACCTGTTTAACTTTGACTG-3′ and antisense 5′-GGACTGGAT GGGATTTAGCCA-3′; NOX2 sense 5′-AGAGGGTTGGAGGTGGAGAATT-3′ and antisense 5′-GCACAAGGAGCAGGACTAGATGA-3′; NOX3 sense 5′-CGTGGCGCATTTCTTCAAC C-3′ and antisense 5′-GCTCTCGTTAGGGGTGTTGC-3′; NOX4 sense 5′-TGTGCCG AACACTCTTGGC-3′ and antisense 5′-ACATGCACGCCTGAGAAAATA-3′; NOX5 sense 5′-CTATTGGACTCACCTGTCCTACC-3′ and antisense 5′-GGAAAAA CAAGAT TCCAGGCAC-3′; CXCR2 sense 5′-CACTCCAATAACAGCAGGTCA-3′ and antisense 5′-AGCAGGCTCAGCAGGAATA-3′; p21 sense 5′-GTCACTGTCTTGTACC CTTGT G-3′ and antisense 5′-CGGCGTTTGGAGTGGTAGAAA-3′; GAPDH sense 5′-CAGAACATCATCCCTGCCTCTAC-3′ and antisense 5′-TTGAAGTCAGAGGAGACCACCTG-3′. Each assay was normalized to the level of GAPDH mRNA.

### Measurement of ROS

ROS generation was measured using oxidation sensitive fluorescent probe (DCFH-DA) according to the manufacturer’s protocols (Beyotime, China). After RSV treatment, cells were harvested and washed with PBS for once, then stained with MDCFH-DA probe at a dilution of 1:2000 in PBS at 37 °C for 20 min. Cells were washed three times with PBS, and the induction of ROS was examined by flow cytometry. In each measurement, 10,000 viable cells were analyzed.

### RNA interference

U2OS cells were transfected with (200 nM) small interfering RNA oligos targeting CXCR2 (5′-GGCAACAAUACAGCAAACT-3′) and a non-silencing scramble RNA duplex (5′-UUCUCCGAACGUGUCA CGU-3′) was used as the negative control. Transfection was performed by using lipofectamine 2000 (Invitrogen) according to the manufacturer's instruction.

### Analysis CXCR2 protein level by flow cytometry

Control and treated cells were harvested using 0.25% Trypsin–EDTA, centrifuged (400xg) for 5 min, and washed once with cold PBS. The cells were incubated with CXCR2 antibody (No. 555751, BD Pharmingen^TM^; 1:6) in 100 μL PBS at room temperature for 30 min. Cells were washed two times with PBS, and the CXCR2 protein level was examined by flow cytometry. In each measurement 10,000 viable cells were analyzed.

### Statistical analysis

All data are presented as mean and SD in triplicate. Student t test was used for comparisons between two groups of experiments. P < 0.05 was considered statistically significant. Statistical significance was also taken as *P < 0.05 and **P < 0.01.

## Electronic supplementary material

Supplementary information
