# Supplementary material for: Resveratrol sequentially induces replication and oxidative stresses to drive p53-CXCR2 mediated cellular senescence in cancer cells
Source: Sci Rep. 2017 Mar 16;7:208. doi: 10.1038/s41598-017-00315-4 (PMC5428242; doi:10.1038/s41598-017-00315-4)
Supplement: Supplementary file 1 — Supplementary information [file 41598_2017_315_MOESM1_ESM.pdf]

Supplementary Information

Title:

Resveratrol sequentially induces replication and oxidative stresses to drive p53-CXCR2 mediated cellular senescence in cancer cells

Author list

Boxuan Li[1], Dong Hou[1], Haiyang Guo[1], Haibin Zhou[1], Shouji Zhang[1], Xiuhua Xu[1], Qiao Liu[1], Xiyu Zhang[1], Yongxin Zou[1], Yaoqin Gong[1], Changshun Shao[1,2,*]

[1]Key Laboratory of Experimental Teratology, Ministry of Education/ Department of Molecular Medicine and Genetics, Shandong University School of Medicine, Jinan, Shandong 250012, China

[2]Department of Genetics/Human Genetics Institute of New Jersey, Rutgers University, Piscataway, NJ 08854, USA

Supplementary Materials

Legends to supplementary figures

Fig.S1. (A) U2OS, NHF, A549 cells were treated with different concentration of RSV for 24 h and then cell cycle distribution was measured by FACS analysis. Data are presented as the mean ± SD of values from triplicate experiments. (B) U2OS, NHF, A549 cells were treated with different concentration of RSV for 7 days. Cellular senescence was examined by SA-beta-gal staining. (C) U2OS and NHF cells were pretreated with the indicated concentrations of RSV for 30min, followed by H_2O_2 challenge for 7 days and then the cells were examined for SA-β-gal staining. Mean of three independent experiments with SEM is shown.

Fig.S2. The cell cycle distribution (A) and ROS levels (B) in HT1080 cells were measured at the indicated time points. Cells were treated with 25 μM RSV for 0, 12, 24, 36, 48h, then the cell cycle distribution and ROS were measured by FACS analysis. (C) The cycle were pretreated with or without 10mM NAC for 1 h, and then followed by 25 μM RSV for 24h or 48 h in HT1080 cells.

Fig.S3. NHFs were maintained in DMEM supplemented with 0.2% FBS for 48h. (A) Cell cycle distribution of serum-starved NHFs measured by FACS analysis. (B) Serum-starved NHFs were treated with the indicated concentrations of RSV for 48 h and ROS was measured by flow cytometry. $^*P < 0.05$. (C) Serum-starved NHFs were treated with the indicated concentrations of RSV for 24 h and stained with mouse anti-γ-H2AX (Ser139). $^*P < 0.05$.

Fig.S4. U2OS (A), NHF (B) and A549 (C) cells were treated with different concentrations of RSV for 48h and then ROS was measured by flow cytometry. Mean of

three independent experiments with SEM is shown. *$P < 0.05$, **$P < 0.01$. (D) U2OS cells were pretreated with the indicated concentrations of RSV for 30 min, followed by H_2O_2 (400 μM) challenge for 3 days and then ROS was measured by flow cytometry. (E) NHF cells were pretreated with the indicated concentrations of RSV for 30min, followed by H_2O_2 (200 μM) challenge for 12h and then ROS was measured by flow cytometry.

Fig.S5. U2OS cells were pretreated with the indicated concentrations of RSV for 30min, followed by a single H_2O_2 (400 μM) treatment, CXCR2 mRNA was determined using real-time RT-PCR 5 days later.

Figure S1

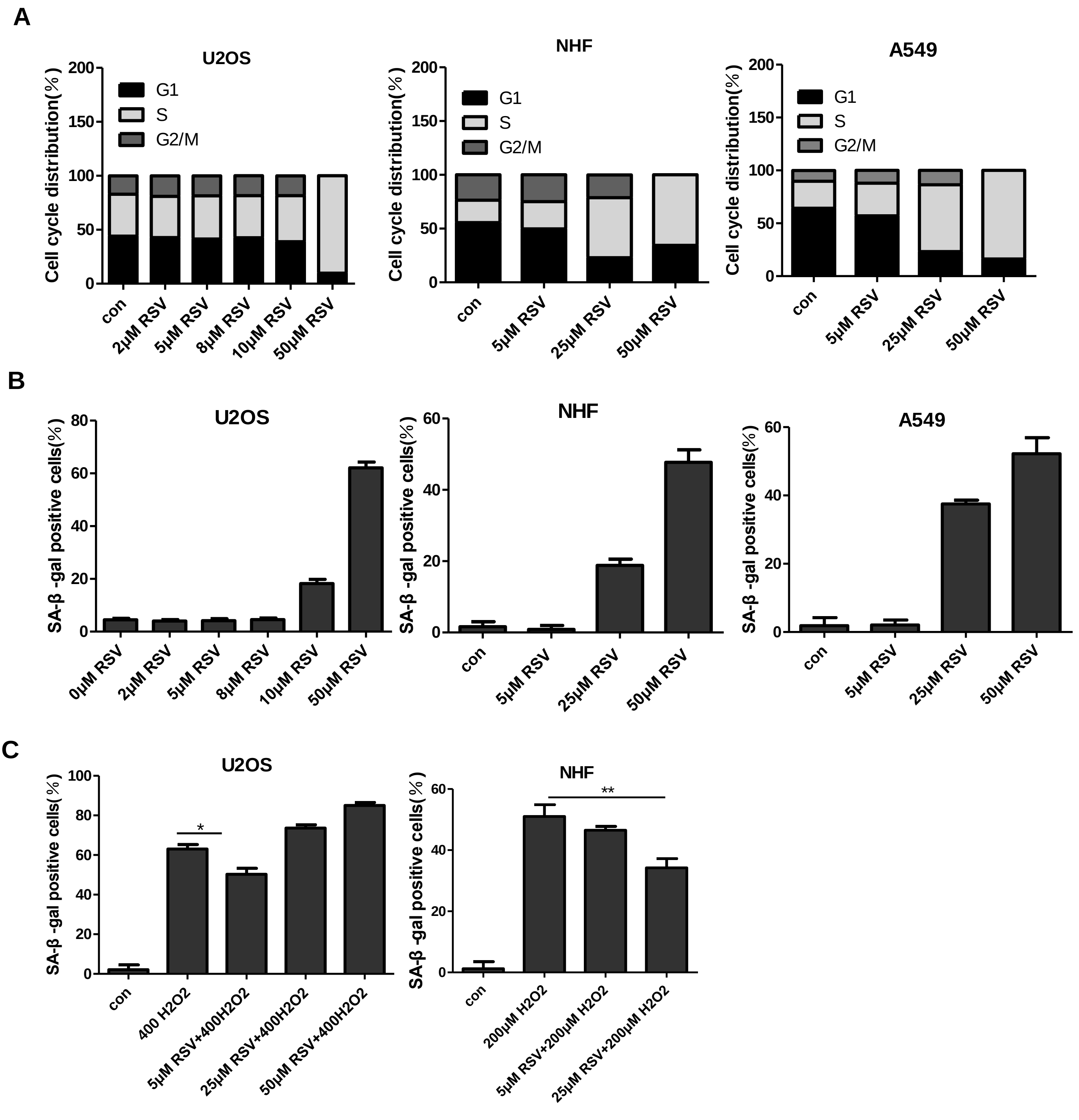
A
U2OS
NHF
A549
Cell cycle distribution(%)
G1
S
G2/M
0
50
100
150
200
con
2μM RSV
5μM RSV
8μM RSV
10μM RSV
25μM RSV
50μM RSV
B
SA-β -gal positive cells(%)
0μM RSV
0
20
40
60
80
C
400 H2O2
5μM RSV+400H2O2
25μM RSV+400H2O2
50μM RSV+400H2O2
200μM H2O2
5μM RSV+200μM H2O2
25μM RSV+200μM H2O2
*
**

Figure S2

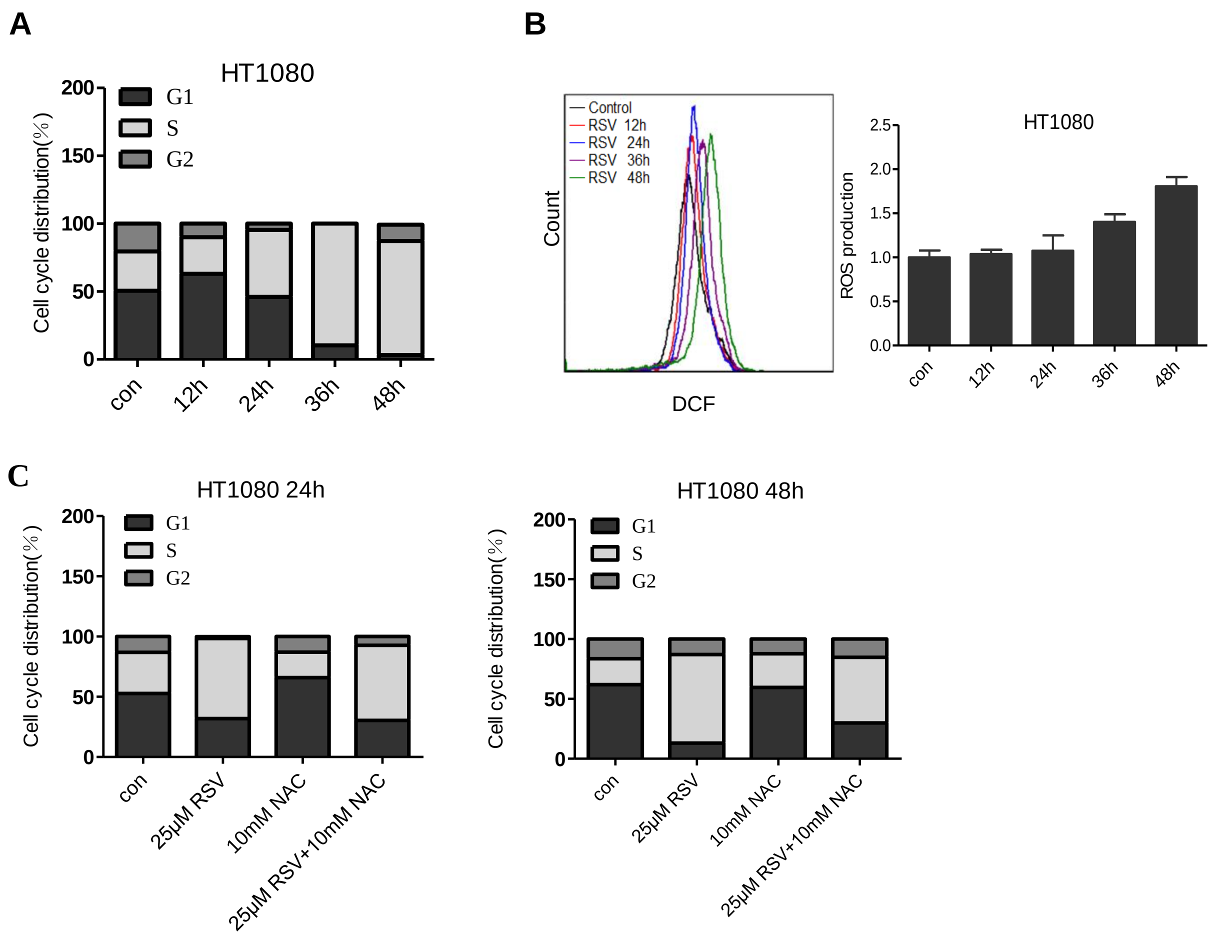
A
HT1080
Cell cycle distribution(%)
G1
S
G2
200
150
100
50
0
con
12h
24h
36h
48h
B
Control
RSV 12h
RSV 24h
RSV 36h
RSV 48h
Count
DCF
HT1080
ROS production
2.5
2.0
1.5
1.0
0.5
0.0
con
12h
24h
36h
48h
C
HT1080 24h
HT1080 48h
Cell cycle distribution(%)
G1
S
G2
200
150
100
50
0
con
25μM RSV
10mM NAC
25μM RSV+10mM NAC

Figure S3

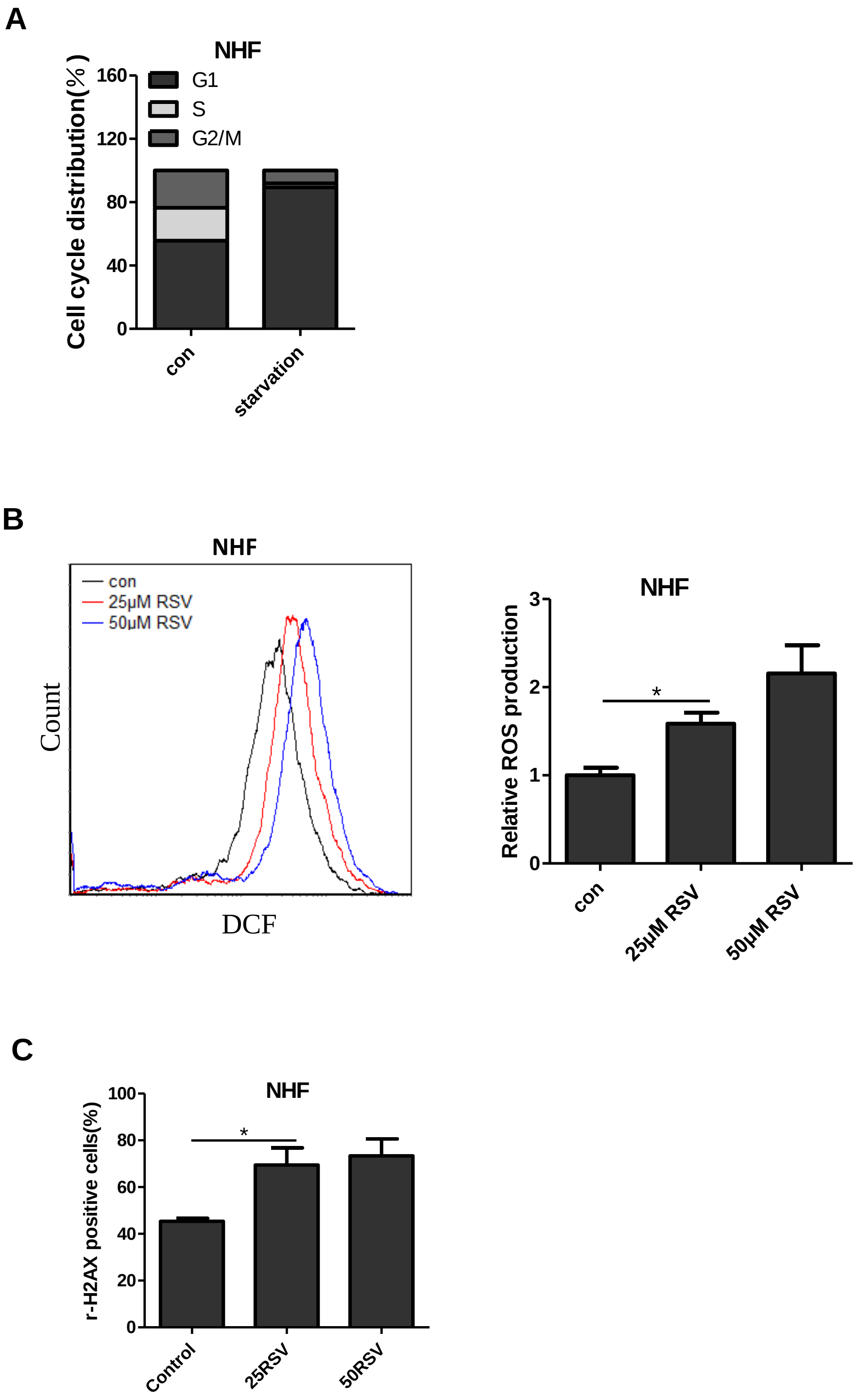
A
NHF
Cell cycle distribution(%)
160
120
80
40
0
G1
S
G2/M
con
starvation
B
NHF
con
25μM RSV
50μM RSV
Count
DCF
NHF
Relative ROS production
3
2
1
0
*
con
25μM RSV
50μM RSV
C
NHF
r-H2AX positive cells(%)
100
80
60
40
20
0
*
Control
25RSV
50RSV

Figure S4

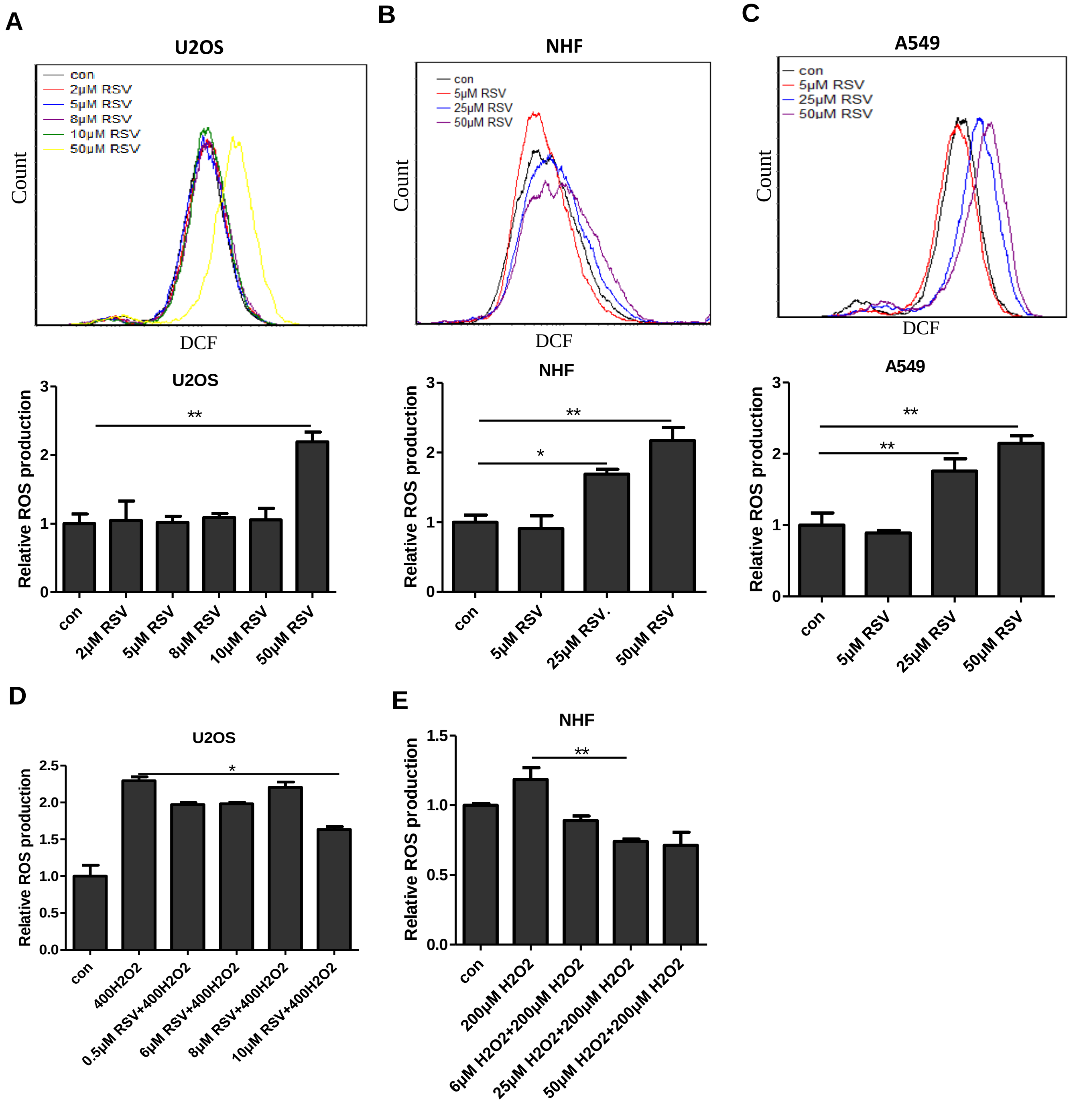

A
U2OS
con
2μM RSV
5μM RSV
8μM RSV
10μM RSV
50μM RSV
Count
DCF
B
NHF
con
5μM RSV
25μM RSV
50μM RSV
Count
DCF
C
A549
con
5μM RSV
25μM RSV
50μM RSV
Count
DCF
U2OS
Relative ROS production
**
con
2μM RSV
5μM RSV
8μM RSV
10μM RSV
50μM RSV
NHF
Relative ROS production
**
*
con
5μM RSV
25μM RSV.
50μM RSV
A549
Relative ROS production
**
**
con
5μM RSV
25μM RSV
50μM RSV
D
U2OS
Relative ROS production
*
con
400H2O2
0.5μM RSV+400H2O2
6μM RSV+400H2O2
8μM RSV+400H2O2
10μM RSV+400H2O2
E
NHF
Relative ROS production
**
con
200μM H2O2
6μM H2O2+200μM H2O2
25μM H2O2+200μM H2O2
50μM H2O2+200μM H2O2

Figure S5

A

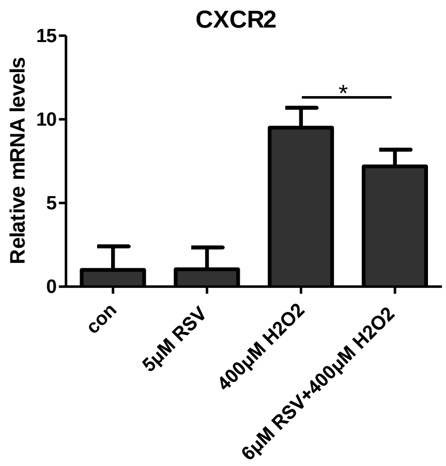

CXCR2
Relative mRNA levels
15
10
5
0
*
con
5μM RSV
400μM H2O2
6μM RSV+400μM H2O2
